# Combining Chronic Ischemic Preconditioning and Inspiratory Muscle Warm-Up to Enhance On-Ice Time-Trial Performance in Elite Speed Skaters

**DOI:** 10.3389/fphys.2018.01036

**Published:** 2018-07-31

**Authors:** Philippe Richard, François Billaut

**Affiliations:** Département de kinésiologie, Université Laval, Quebec, QC, Canada

**Keywords:** warm up, chronic ischemic preconditioning, high-level athletes, muscle oxygen extraction, blood volume, sprint

## Abstract

Elite athletes in varied sports typically combine ergogenic strategies in the hope of enhancing physiological responses and competitive performance, but the scientific evidence for such practices is very scarce. The peculiar characteristics of speed skating contribute to impede blood flow and exacerbate deoxygenation in the lower limbs (especially the right leg). We investigated whether combining preconditioning strategies could modify muscular oxygenation and improve performance in that sport. Using a randomized, single-blind, placebo-controlled, crossover design, seven male elite long-track speed skaters performed on-ice 600-m time trials, preceded by either a combination of preconditioning strategies (COMBO) or a placebo condition (SHAM). COMBO involved performing remote ischemic preconditioning (RIPC) of the upper limbs (3 × 5-min compression at 180 mmHg and 5-min reperfusion) over 3 days (including an acute treatment before trials), with the addition of an inspiratory muscle warm-up [IMW: 2 × 30 inspirations at 40% maximal inspiratory pressure (MIP)] on the day of testing. SHAM followed the same protocol with lower intensities (10 mmHg for RIPC and 15% MIP). Changes in tissue saturation index (TSI), oxyhemoglobin–oxymyoglobin ([O_2_HbMb]), deoxyhemoglobin–deoxymyoglobin ([HHbMb]), and total hemoglobin–myoglobin ([THbMb]) in the right vastus lateralis muscle were monitored by near-infrared spectroscopy (NIRS). Differences between COMBO and SHAM were analyzed using Cohen’s effect size (ES) and magnitude-based inferences. Compared with SHAM, COMBO had no worthwhile effect on performance time while mean Δ[HHbMb] (2.7%, ES 0.48; -0.07, 1.03) and peak Δ[HHbMb] (1.8%, ES 0.23; -0.10, 0.57) were respectively *likely* and *possibly* higher in the last section of the race. These results indicate that combining ischemic preconditioning and IMW has no practical ergogenic impact on 600-m speed-skating performance in elite skaters. The low-sitting position in this sport might render difficult enhancing these physiological responses.

## Introduction

Elite speed skaters adopt a crouched position that is both aerodynamically and biomechanically favorable to performance (Noordhof et al., 2014). However, the combination of this low-sitting position, the isometric gliding phase and high intramuscular forces results in impeded blood flow to working muscles (Konings et al., 2015). Near-infrared spectroscopy (NIRS) studies further demonstrated that a low-skating position during in-line speed skating is associated with an accentuated deoxygenation when compared to an upper-skating position (Rundell et al., 1997). Comparison of oxygenation patterns in short- vs. long-track speed skating demonstrates that the former discipline leads to a more severe muscle deoxygenation (Hettinga et al., 2016). Moreover, higher perceived fatigue and slower recovery are reported two and four hours after short- vs. long-track time trials (Hettinga et al., 2016), suggesting that a greater deoxygenation in active muscles may negatively influence muscle recovery processes. Importantly, blood flow occlusion and tissue deoxygenation also occur during the gliding and push-off phases in long-track speed skating (Hettinga et al., 2016), thereby implicating physiological drawbacks, such as accentuated local hypoxic stress that may hasten peripheral fatigue development (Konings et al., 2015). The exact impact of the deoxygenation severity during speed-skating performance is however not clearly understood.

In order to optimize energy metabolism and with the ultimate goal of achieving maximal performance, elite athletes of different sports adopt several preconditioning techniques (Kilduff et al., 2013), priming exercises (Bailey et al., 2009) and warm-up strategies (McGowan et al., 2015). Although some of these protocols were found to modify oxygenation and improve performance, very few of these techniques were investigated on elite speed skaters during a specific performance task. Furthermore, data on the impact of a combination of techniques are very scarce in the literature.

In various sports and research settings, remote ischemic preconditioning (RIPC) was found to enhance performance [time to task failure (Barbosa et al., 2015; Kido et al., 2015; Tanaka et al., 2016), peak and mean power output (Patterson et al., 2015), maximal concentric force (Paradis-Deschênes et al., 2016)] concomitantly with an altered deoxygenation [attenuated (Kido et al., 2015; Patterson et al., 2015), accentuated (Barbosa et al., 2015; Paradis-Deschênes et al., 2016), and accelerated dynamics (Kido et al., 2015; Tanaka et al., 2016)]. These data highlight the equivocal relationship between oxygenation alterations and performance. Interestingly, our research group investigated the effects of RIPC on a 1000-m speed-skating race. This technique did not enhance performance, but was associated with an accentuated deoxygenation in sprint-specialized speed-skaters (Richard and Billaut, 2018). Considering the high ischemic stress associated with speed-skating itself and the fact that elite athletes present a narrower window of adaptation compared to less trained individuals (Marocolo et al., 2016a), we reasoned that a chronic RIPC stimulation (Foster et al., 2014) could represent a more effective strategy to modify acute physiological response and performance in a population of elite speed skaters.

An inspiratory muscle warm-up (IMW) was found to improve performance and lower lactate concentration in badminton players during specific footwork (Lin et al., 2007) and to significantly attenuate NIRS-derived deoxygenation (tissue saturation index, TSI) of the gastrocnemius muscle during submaximal and high-intensity intermittent sprint cycling exercises in elite female soccer players (Cheng et al., 2013). Moreover, the combination of a specific rowing warm-up with IMW led to a significant improvement in a 6-min all-out rowing effort compared to a specific rowing warm-up alone (Volianitis et al., 2001). However, IMW has not been investigated in a sprint speed skating race, notwithstanding this technique was found to improve 100-m race in elite swimmers (Wilson et al., 2014), Wingate test in field hockey players (Özdal et al., 2016), and intermittent running performance in healthy males (Tong and Fu, 2006).

Therefore, considering the potential cumulative impact of such techniques, the fact that elite athletes combine different methods, and in the spirit of answering a specific research question that was raised in preparation for the Olympic trials and competitions, the primary purpose of this study was to examine whether the combination of chronic RIPC with IMW could improve performance and modify muscular oxygenation during a 600-m speed-skating time-trial in elite speed skaters.

## Materials and Methods

### Participants

Seven male elite long-track speed skaters (four with senior world cups and/or world championship experience, two with international junior championship experience, and one national level speed skater) volunteered for this study (age 23.4 ± 3.3 years, body height 181.79 ± 7.19 cm, and body mass 80.91 ± 7.72 kg). The limited number of high-level athletes available for this project is the main reason for the small sample size in this study. The athletes had 16 ± 6 years of experience in speed skating (short and long track), and their average weekly training volume was ∼13–15 h per week at the time of the study. The investigation took place during the training season and, therefore the timed-performances in the present study do not reflect the best potential results of the athletes. Personal best (Pb) for 500- and 1000-m races are presented as an indication of the level of the skaters (Pb500m: 35.26 ± 1.11 and Pb1000m: 69.19 ± 1.73). All participants provided written informed consent after being informed of the experimental procedures, associated risks, and potential benefits. The team physician approved the participation of the athletes for this research project. The study was approved by the local Institutional Ethics Committee (*Comité d’éthique de la Recherche en Sciences de la Santé*) and by the local Hospital Ethics Committee (*Comité d’éthique de la Recherche de l’IUCPQ-Université Laval*), and in accordance with the principles established in the Declaration of Helsinki.

### Experimental Design

All athletes were tested on two occasions in a randomized, single-blind, placebo-controlled, and crossover design. Specifically, the participants were blinded about the impact of the interventions, but the tester was aware of which protocol the subjects were undergoing and had undergone before. The athletes were blinded about their physiological, technical (push-off angle) and transponders (maximal velocity) data until the end of the research. However, to promote a realistic context, the athletes were not blinded about their performance (timing system) data. Athletes did not consume any caffeine, drugs, or supplements for 24 h before the tests and had a similar competition-specific diet before the two trials. All athletes participated in a maximal inspiratory pressure (MIP) testing session, were fully familiarized with IMW, RIPC, and SHAM procedures at least 14 days before the first race and the order of the two trials was interspersed with 7 days.

Maximal inspiratory pressure was recorded using the integrated mouth pressure meter of the Vmax ENCORE system (CareFusion) at the local hospital. The testing session was performed by an experienced professional operator and in conformity to standardized procedures (Gibson et al., 2002). Hans Rudolph mouthpieces standard type (clear) reusable series 9060 were used for the testing. A minimum of five and a maximum of nine technically satisfactory measurements were conducted, and the highest of three measurements with 5% variability or within 5 cmH_2_O difference was defined as maximum (Volianitis et al., 2001). The initial length of the inspiratory muscles was controlled by initiating each effort from residual volume. Verbal encouragement was given to assist the subjects perform maximally.

To minimize any placebo effect, participants were told that the study purpose was to compare the impact of two different combinations of preconditioning strategies (high intensity: RIPC_highpressure_ + IMW_strength_ or low intensity: RIPC_lowpressure_ + IMW_endurance_) that could either alter arterial inflow and IM strength (COMBO) or micro perfusion and IM endurance capacity (SHAM). They were told that both combinations could potentially alter performance positively as follows: “*the aim of the study was to determine the best suitable combination of intervention individually for each of them*.”

### Chronic Remote Ischemic Preconditioning

Remote ischemic preconditioning treatment implicated three alternating 5-min cycles of upper-limb compression interspaced with 5-min of reperfusion. The occlusion pressure was set at 180 mmHg (Lisbôa et al., 2017) in COMBO (mean systolic pressure: 116.7 ± 7.9 mmHg). The SHAM treatment followed the exact same procedure with a given pressure of 10 mmHg. In both conditions, participants were lying on their back on a massage table and one nylon blood pressure cuff (Welch Allyn, Skaneateles Falls, NY, United States or Almedic, Saint-Laurent, Montreal, QC, Canada) was positioned proximally around each arm. The size of the cuff was chosen in accordance to arm circumference, in respect to manufacturer instructions. Upper limbs were chosen for the treatment as the remote stimulation was shown to have a systemic vasoactive effect (Enko et al., 2011) and to alter performance (Barbosa et al., 2015). Furthermore, the large lower-body muscular mass and thigh circumference of elite speed skaters would necessitate a very high pressure to induce complete arterial blood flow blockage (Sharma et al., 2014) and, therefore, could implicate higher levels of subjective pain and a potential nocebo effect (Salvador et al., 2015). Both interventions (RIPC and SHAM) were conducted ∼48, ∼24, and ∼1.5 h before the trials (the last compression cycle occurred ∼60-min before the race). This chronic and acute RIPC protocol allowed the skaters to benefit from both the second window of conditioning (Yellon and Downey, 2003; Berger et al., 2015) and the early phase of conditioning (Yellon and Downey, 2003; Berger et al., 2015). It also allowed sufficient time to warm up (including IMW) and prepare according to usual competitive habits.

### Inspiratory Muscle Warm-Up

The protocol consisted of two sets of 30 breaths using a POWERbreathe (IMT Technologies Ltd., Birmingham, United Kingdom) at 40 and 15% MIP in IMW and SHAM, respectively, with a 60 s rest between sets (Lomax et al., 2011). Previous studies have indicated that specific IMW protocol at a given intensity of 40% MIP improves performance (Tong and Fu, 2006; Lin et al., 2007; Wilson et al., 2014). During the IMW trial, the subjects were instructed to initiate every breath from the residual volume and to continue the respiratory effort up to the lung volume where the IM force output for the given load limited further excursion of the thorax. During the SHAM trial, the breaths were performed gently and the respiratory time for each breath was protracted (Ohya et al., 2015). Both interventions were conducted only on racing days, after the athlete’s off-ice warm-up, ∼20-min before the time trials.

### Warm-Up

To promote a realistic context, the athletes were asked to reproduce their regular individual competition warm-up routine. However, the athletes awareness was raised on the fact that priming exercises can potentially influence peripheral oxygenation (Jones et al., 2006; Bailey et al., 2009; McIntyre and Kilding, 2015) in a similar way as the studied interventions (Salvador et al., 2015). Precisely, athletes were told that omitting to perform intensity bouts in their warm-ups 20–40-min before their race (Burnley et al., 2006; Bailey et al., 2009; Ingham et al., 2013) could potentially negatively affect their performance (Bailey et al., 2009) and mislead conclusion about the current research. Post-testing, all athletes reported having integrated priming exercises (∼30-min before the race) in their off-ice warm-up as well of having reproduced a very similar warm-up in both conditions (**Table 1**).

**Table 1:** Individual warm-up routine characterization including priming exercises and recovery time before the race for all speed skaters.

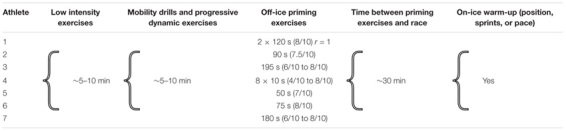			

### Speed Skating Measurements

#### Time-Trials and Performance Measurements

The trials involved two 600-m race simulations on an indoor long-track (400-m) speed-skating oval approved for international competition. This distance was chosen in order to be able to compare the results of the present study (opener: 200-m + lap1: 400-m = 600-m) with that of our previous speed-skating-RIPC investigation (opener: 200-m + lap1: 400-m + lap2: 400-m = 1000-m) (Richard and Billaut, 2018). The 1000-m time-trial was avoided to prevent the athletes from experiencing high-level of fatigue and to limit interference with training. Trials took place during a national center training camp in which the training prescription was similar for the 48 h before each trial. Environmental conditions were noted in both testing days (barometric pressure: 88.79 vs. 88.30 kPa, ice temperature: -5.5° vs. -5.2°, average ambient temperature: 15.8° vs. 14°). In both conditions, the athletes wore the same skin suit, started in the inner lane, and were asked to complete the 600-m in the fastest time possible. Verbal feedback was given by the coach to ensure competition-like conditions (Born et al., 2014). Depending on the stage of preparation of the athletes, it was at some occasions, possible for the other participants to see their teammates performed the test. However, the order of the tests and the chronology of the preparation of the athletes were the exact same in both conditions. During the races, all lap and split times we recorded using a timing system approved for international competition. Athletes were also wearing transponders approved for international competition (MYLAPS, Nijmegen, Netherlands) around each ankle to permit velocity measurements in different sections of the race.

#### Technical Measurements

The effectiveness of the push-off (direction of the push-off force) is reflected by the angle *e* [push-off angle (*e*): the angle between the push-off leg and the horizontal] (Noordhof et al., 2014). Skating efficiency is reflected by smaller push-off angle and previous studies reported this angle as one of the key performance-determining variables (Noordhof et al., 2013). Therefore, participants were filmed from a frontal view in the first straight (inner lane) during both testing conditions with one digital high-definition camera (Canon, VIXIA HF R50, Tokyo, Japan). The camera was placed at the end of the inner lane straight, in the middle of the lane and tripod height was constant, in both conditions. The push-off angle was measured for the third, fourth, eighth, and ninth strides of the first straight using video-based movement analysis software (Dartfish). The frame approximately before the moment the hinge of the klapskate of the push-off leg opened was used to determine *e* for each stride. The average of theses four angles was deemed as the *e* for this section of the race. A correction for a slightly skewed camera position was made to the calculated *e* using the vertical coordinates of a horizontal marker that was present behind the skaters in the analyzed section (Noordhof et al., 2013).

### Physiological Measurements

#### NIRS Measurements

Oxygenation patterns in the right vastus lateralis muscle were determined with a wireless portable NIRS device (PortaMon MkII, Artinis Medical Systems, Zetten, Netherlands). Bilateral oxygenation measurements would have provided a more complete dataset about the effects of the techniques investigated here considering the asymmetric oxygenation patterns reported in speed skating (Born et al., 2014; Hettinga et al., 2016). However, only one PortaMon device was available for this project. The right leg was particularly investigated as long-track speed skaters display a greater deoxygenation in the right leg compared to the left leg (Born et al., 2014; Hettinga et al., 2016). Investigating the right leg oxygenation profile also allows data comparison with previous studies on priming exercises (Bailey et al., 2009), IPC/RIPC (Paradis-Deschênes et al., 2017), IMW (Ohya et al., 2015), and slide board skating (Piucco et al., 2017).

The NIRS device was installed on the distal part of the vastus lateralis belly (15 cm above the proximal border of the patella). Skin fold thickness was measured at the site of application of the NIRS device (7.7 ± 2.5 mm) using a Harpenden Skinfold Caliper (Harpenden Ltd.) during the familiarization session, and was less than half the distance between the emitter and the detector (i.e., 20 mm). This thickness is adequate to let near-infrared light through muscle tissue (McCully and Hamaoka, 2000). The device was fixed using tape and covered with black bandages and the speed-skating skin suit to eliminate background light. Due to the influence of the site of investigation and adipose tissue thickness on the recorded NIRS parameters (Van Der Zwaard et al., 2016), the device position was marked with an indelible pen for the subsequent trial.

A modified form of the Beer–Lambert law, using two continuous wavelengths (760 and 850 nm) and a differential optical path length factor of 4.95 was used to calculate micromolar changes in tissue oxyhemoglobin–oxymyoglobin ([O_2_HbMb]), deoxyhemoglobin–deoxymyoglobin [HHbMb], and total hemoglobin–myoglobin ([THbMb]) ([THbMb] = [O_2_HbMb] + [HHbMb]), which is used as an index of change in regional blood volume (Van Beekvelt et al., 2001). The equilibrium between oxygen supply and consumption was calculated using the TSI (%) as [HbO_2_Mb] divided by ([O_2_HbMb] + [HHbMb]) × 100. The [HHbMb] signal was also taken as an indicator of tissue deoxygenation because this variable is less sensitive than [O_2_HbMb] to perfusion variations and abrupt blood volume changes during contraction and recovery (Van Beekvelt et al., 2002; Ferrari et al., 2004; Grassi et al., 2007).

Data were acquired continuously at 10 Hz. A 10th order zero-lag low-pass Butterworth filter was applied to smooth NIRS signal (Faiss et al., 2013). Data were analyzed over the first 15 s (START) and between 15 and 40 s (END) as similarly described in a previous speed-skating investigation (Born et al., 2014) to allow for the comparison of oxygenation variables over effort of equal duration, and normalized to express the magnitude of changes from baseline.

#### Heart Rate Measurements

Heart rate (HR) was monitored during racing using the athlete’s personal monitoring devices: Polar M 400 (Polar Electro, Kempele, Finland) and Garmin Forerunner 920XT (Garmin, KS, United States). Average HR could not be extracted because of technical problems; however, max HR was collected for six of the seven athletes.

### Perceptual Measurements

Immediately after the races, rate of perceived exertion and rate of perceived breathlessness were measured using CR-10 Borg’s scale (Ferreira et al., 2016). Expected benefit was also measured by asking athletes to rate their general expectation for both conditions considering their individual preparation, warm-up, readiness and the studied interventions on a scale from 1 to 10.

### Statistical Analyses

COMBO–SHAM differences were analyzed using Cohen’s effect size (ES) ± 90% confidence limits, and magnitude-based inferences (Hopkins et al., 2009). We used this qualitative approach because traditional statistical approaches often do not indicate the magnitude of an effect, which is typically more relevant to athletic performance than any statistically significant effect (Hopkins et al., 2009). All variables were log-transformed before analysis (Hopkins et al., 2009), but raw data are reported as means or peaks ± SD for clarity. Magnitudes of difference between conditions were determined with an ES of 0.2 set to evaluate the smallest worthwhile change. Standardized effects were classified as small (>0.2), moderate (>0.5), or large (>0.8). Quantitative chances of greater or smaller values were assessed qualitatively as follows: 50 to 75%, possibly; 75 to 95%, likely; 95 to 99%, very likely; >99%, almost certainly. The effect was deemed “unclear” if chances of having better/greater and poorer/lower change in performance and physiological variables were both >5% (Hopkins et al., 2009).

## Results

All participants met all the criteria for inclusion, and tolerated the RIPC and IMW procedure without complications. All participants completed the entire testing protocol (i.e., 2 × 600-m).

### Speed Skating Performance and Technical Variables

**FIGURE 1:**
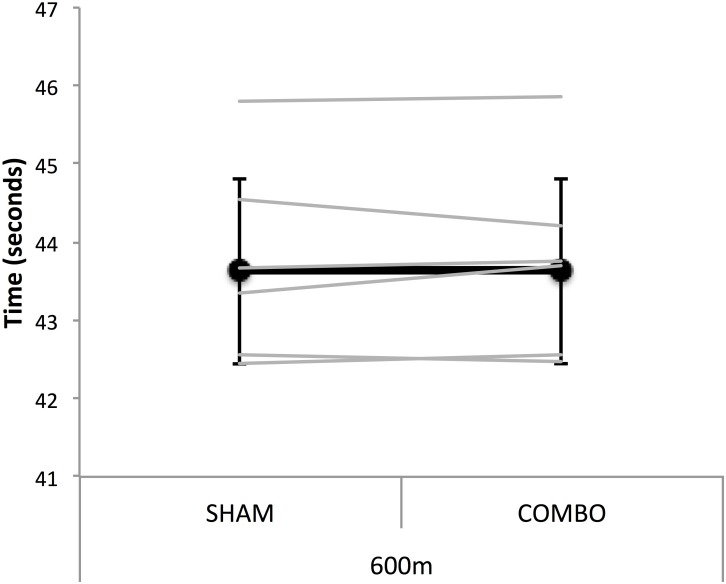
Mean (black line) and individual (gray lines) performance time for the 600-m race simulation in SHAM and COMBO conditions. Data are presented as mean ± SD.

Individual and group mean performances are displayed in **Figure 1** and **Table 2**. COMBO had no clear effect on either overall performance (0.06% mean difference, ES 0.02; 90% confidence limits -0.09, 0.12) or pacing strategy. There was no clear effect of the intervention on maximal velocity measured with transponders in the first 200-m (54.20 ± 1.94 vs. 54.23 ± 2.16 km/h: 0.04% mean difference, ES 0.02; -0.24, 0.26) and in the last 400-m of the race (55.18 ± 1.51 vs. 55.30 ± 1.40 km/h: 0.21% mean difference, ES 0.07; -0.06, 0.19). Push-off angle (*e*) in the first straight also remained unaffected by the intervention (43.74 ± 1.75 vs. 43.80 ± 2.05°: 0.11% mean difference, ES 0.02; -0.17, 0.22).

**Table 2:** Physiological and performance measures for the on-ice 600-m time-trial.

Variable	Time point	Intervention						Likelihood of chances
		SHAM	SD	COMBO	SD	*d*	CL	+ve/trivial/-ve
Tissue saturation index (TSI%)	0–15 s	44.32	2.62	44.45	3.05	0.04	-0.47; 0.55	28/52/20
	15–40 s	39.35	3.26	38.58	4.4	-0.33	-1.05; 0.38	10/27/63
Tissue saturation index (TSI%)/baseline (% baseline)	0–15 s	75.1	3.8	75.4	5.8	0.04	-0.55; 0.64	31/46/23
	15–40 s	66.6	4.5	65.4	5.8	-0.39	-1.23; 0.45	11/23/66
Average deoxyhemoglobin (HHbMb)	0–15 s	48.45	8.1	48.86	8.56	0.03	-0.04; 0.11	0/100/0
	15–40 s	54.86	9.5	56.37	9.83	0.12	-0.02; 0.27	17/82/0
Average deoxyhemoglobin (HHbMb)/baseline (% baseline)	0–15 s	113.8	5.4	114.6	5.5	0.13	-0.17; 0.43	33/63/4
	15–40 s	128.9	9.5	132.3	8.4	0.48	-0.07; 1.03	82/15/3
Peak deoxyhemoglobin (HHbMb)	0–15 s	58.86	10.39	59.16	10.52	0.02	-0.15; 0.19	5/93/2
	15–40 s	60.9	10.92	62.11	11.87	0.08	-0.03; 0.19	4/96/0
Peak deoxyhemoglobin (HHbMb)/baseline (% baseline)	0–15 s	138.2	9	138.8	7.5	0.07	-0.45; 0.58	32/51/18
	15–40 s	142.8	8.6	145.4	9.9	0.23	-0.10; 0.57	58/40/2
Oxyhemoglobin (O2HbMb)	0–15 s	36.17	4.16	36.9	4.07	0.16	-0.18; 0.49	40/55/4
	15–40 s	34.18	4.34	35.25	5.15	0.22	-0.27; 0.71	53/40/7
Oxyhemoglobin (O2HbMb)/baseline (% baseline)	0–15 s	66.8	5.4	68.1	4.4	0.21	-0.24; 0.66	52/42/6
	15–40 s	63.1	4.7	64.9	5.4	0.29	-0.36; 0.95	60/30/10
Total hemoglobin (THb)	0–15 s	84.62	11.55	85.76	12.14	0.08	-0.08; 0.24	10/90/1
	15–40 s	89.04	12.77	90.11	13.12	0.07	-0.07; 0.22	7/93/1
Total hemoglobin (THb)/baseline (% baseline)	0–15 s	87.4	3.3	88.5	2.4	0.29	-0.30; 0.88	61/31/8
	15–40 s	91.9	3.6	92.9	2.6	0.26	-0.27; 0.79	59/34/7
Maximal heart rate, beats/min	During 600 m	189.3	7.36	189.3	9.09	-0.01	-0.26; 0.25	8/82/10
Push-off angle (*e*),°	First straight	43.74	1.75	43.8	2.05	0.02	-0.17; 0.22	6/90/3
Maximal velocity, km/h	First 200-m	54.2	1.94	54.23	2.16	0.01	-0.24; 0.26	9/83/8
	Last 400-m	55.18	1.51	55.3	1.4	0.07	-0.06; 0.19	4/96/0
Time (performance), seconds	200-m	17.19	0.55	17.23	0.6	0.08	-0.08; 0.24	10/89/1
	600-m	43.63	1.19	43.63	1.19	0.02	-0.09; 0.12	1/99/0

### Physiological Variables

Maximal HR remained unaffected by the intervention (189.83 ± 7.36 vs. 189.83 ± 9.09 beats/min: -0.03% mean difference, ES -0.01; -0.26, 0.25).

**FIGURE 2:**
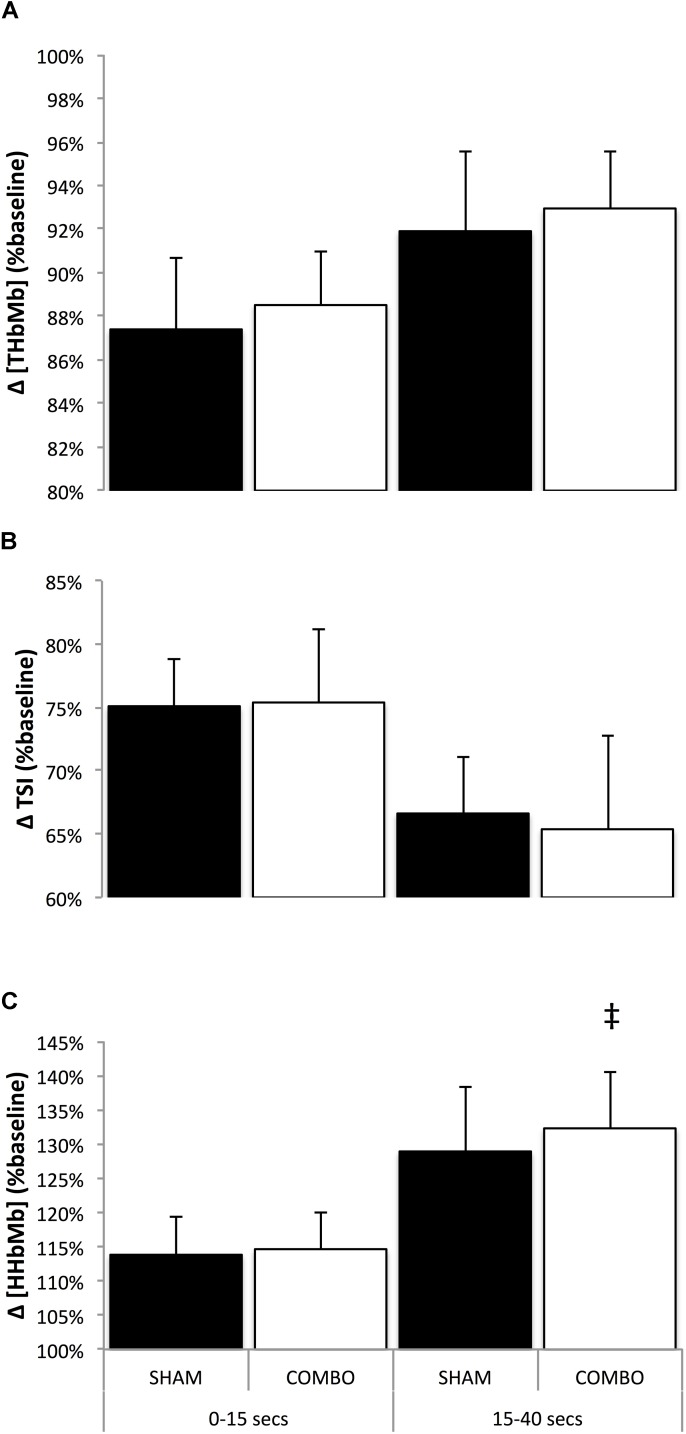
Mean changes in [THbMb] **(A)**, TSI **(B)**, and [HHbMb] **(C)** for the first 15 s (0–15 s), and from second 15 to second 40 (15–40 s) in SHAM and COMBO conditions. Black bars (SHAM) and white bars (COMBO). Data are presented as mean ± SD. ^‡^Likely clear effect in COMBO vs. SHAM.

There was no clear effect of the intervention on TSI, Δ[O_2_HbMb] and Δ[THbMb] for both analyzed sections of the race. Mean and peak Δ[HHbMb] were also unaffected by the COMBO in the START section of the trial, however, mean Δ[HHbMb] was *likely* higher in the END section of the race (2.7%, ES 0.48; -0.07, 1.03) and peak Δ[HHbMb] was *possibly* higher in that same section (1.8%, ES 0.23; -0.10, 0.57) in COMBO compared with SHAM (**Table 2** and **Figure 2**).

### Perceptual Measures

Post-facto interviews completed at the end of the project revealed that none of the athletes knew there was a PLACEBO intervention indicating they were all confounded by the true objective of the study. Interestingly however, expected perceived benefits were *possibly* higher (4.1%, ES 0.35; -0.22, 0.91) in COMBO (7.71/10 ± 0.57) compared to SHAM (7.43/10 ± 0.79), but no difference were observed in rate of perceived exertion (SHAM: 8.36/10 ± 0.63, COMBO: 8.43/10 ± 0.61) and rate of perceived breathlessness (SHAM: 8/10 ± 0.65, COMBO: 7.86/10 ± 1.18) between conditions.

## Discussion

The primarily purpose of this investigation was to test the potential ergogenic impact of a combination of preconditioning strategies, that were previously found to enhance performance in predominantly anaerobic oriented exercises (Wilson et al., 2014; Salvador et al., 2015; Özdal et al., 2016), on a 600-m time-trial in elite speed skaters. This study also examined the impact of the combination of RIPC and IMW, two preconditioning strategies that were found to modify peripheral oxygenation (Cheng et al., 2013; Kido et al., 2015; Tanaka et al., 2016) in some context, on the vascular and metabolic effects induced by the crouched position adopted by elite speed skaters in race. Considering the certain level of permeability that we previously observed in elite speed skaters regarding acute RIPC (Richard and Billaut, 2018), and since elite athletes present a narrower window of adaptation compared to less trained individuals (Marocolo et al., 2016a) and tend to combine different methods to enhance their performances (Kilduff et al., 2013; McGowan et al., 2015), we reasoned that the aggregation of such techniques could be necessary to trigger an ergogenic response and to enhance performance in this population. However, the current data showed no performance-enhancing effect (split time, final time, and velocities) of COMBO on an all-out 600-m speed-skating time trial lasting ∼43 s, and indicated that the addition of this intervention to an elite long-track skater’s competitive warm-up routine is insignificant in regard to performance. Furthermore, skating efficiency, which is reflected by push-off angle (*e*) (Noordhof et al., 2013) was not affected by combo in the analyzed section of the race (first straight). Besides, although most of the physiological data were negligibly influenced by COMBO, mean and peak Δ[HHbMb] were respectively *likely* and *possibly* increase in the END portion of the race, which may presumably be related to a higher O_2_ extraction at the muscular level.

### Preconditioning Strategies in Elite Sprint Speed Skaters

This investigation that took place during the training season revealed that a combination of preconditioning strategies does not improve speed skating 600-m time-trial performance. The current findings contrasts with the results of a meta-analysis on the effect of IPC on performance that reported a small but clear effect of this intervention (ES 0.23) on exercises lasting 10–90 s (Salvador et al., 2015). The present findings also contrast with studies reporting an ergogenic effect of IMW on a 100-m swimming performance (∼75 s) and on a Wingate test (Özdal et al., 2016). Therefore, we propose that the peculiar characteristics of speed skating (Konings et al., 2015; Hettinga et al., 2016), the high level of the athletes included in our study or a mix of these two factors, may contribute to explaining our findings.

On the one hand, Salvador et al. (2015) found no evidence for a greater benefit of IPC in less fit individuals compared to athletes in their meta-analysis. However, the current available literature contains very few studies investigating the effect of IPC/RIPC in high-level elite athlete populations (Incognito et al., 2015). In that regard, it has been suggested that highly trained subjects are expected to have high NOS activity (McConell et al., 2007). This higher skeletal muscle NOS protein expression (nNOSμ) is likely to be associated with greater production of NO by skeletal muscle, which might render this population less dependent on the NO pathway (McConell et al., 2007). NO seems to be a key factor in the mechanistic response to RIPC (Incognito et al., 2015), thereby potentially minimizing the effect of such an intervention in an elite athlete population (Richard et al., 2018). On the other hand, the two above-mentioned IMW studies included respectively high-level swimmers and field hockey players, which contribute to making the comparison easier with our study. Nevertheless, the fact that swimming, a sport that presents several unique challenges to the respiratory system (Wilson et al., 2014), was investigated in the former of theses two investigations, and the fact that the latter IMW study was not placebo-controlled (Özdal et al., 2016) may naturally contribute to explaining theses positive outcomes. Besides, the potential ergogenic effect of COMBO might also have been impeded completely or in part by the sport-specific reduced blood flow associated with the low skating position, the relatively long gliding phase and the high intramuscular forces specific to speed skating (Konings et al., 2015). Therefore, based on the available data, it can be misleading to speculate whether the level of the athletes, the characteristics of the sport, or the combined efficiency of the tested techniques may be deemed as the main reason of absence of ergogenic impact observed in our study.

### Expected Benefits and Performance

Although the athletes were (*possibly*) expecting greater benefits in COMBO than in SHAM condition (considering their global preparation, including the interventions), these higher expectations (ES: 0.35) did not translate into an improved performance. In fact, it is worth highlighting that, after performing their individual competitive warm-up, both COMBO and SHAM yielded an identical time of 43.63 ± 1.19 s. The results of other investigations suggest that, the effect of IPC does not surpass a placebo intervention and/or that the ergogenic effect associated with IPC may be mainly attributed to a placebo effect (Marocolo et al., 2015; Succi, 2016). After the study, when we debriefed the athletes in regard to the true SHAM interventions, none of them were aware of the presence of any placebo procedures. Therefore, their higher expectation may have been mainly related to their general preparation rather than to the COMBO *per se*. Another interesting finding is that both IPC and SHAM interventions led to performance improvements on a resistance exercise test (∼40 s, such as in the present study), but these ergogenic effects faded over time (over only four trials) (Marocolo et al., 2016b). While these results suggests that performance improvement after IPC (or SHAM) may mainly be attributable to motivational issues, the fact that these ergogenic effects tend to fade away with time decreases the practical value of such interventions in an elite sport setting where athletes compete on a regular basis.

### Accentuated Deoxygenation

Although performance was unaffected by COMBO, muscle deoxygenation was accentuated (higher mean and peak [HHbMb]) in the END section of the race. Interestingly, a similar enhanced [HHbMb] response was observed after acute RIPC, only in sprint specialized skaters, during a 1000-m speed skating time trial (Richard and Billaut, 2018). This response was observed during the entire 1000-m race (∼75 s) and was concomitant with a possible increase in blood volume ([THbMb]) in the middle section of the race only. Taken these data together, it can be hypothesized that the analogous ([HHbMb]) response observed in the present investigation occurred because most of the athletes included in the study were sprint-specialized skaters. Indeed, when further studying the specific response of the four sprint-specialized skaters of international senior level (Pb 500-m, 34.45 ± 0.3, Pb 1000-m: 67.97 ± 0.36) in a post-facto analysis, we observed a likely enhanced ([HHbMb]) in the END section of the race (mean [HHbMb]: 3.6%, ES 0.56; -0.20, 1.33 and peak [HHbMb]: 2.7%, ES 0.42; -0.18, 1.01) that was greater than the whole group response. However, in the present study, this greater O_2_ extraction was not accompanied by a clear effect on [THbMb], a surrogate of blood volume, either for the whole group or the four international level sprinters. In the aforementioned RIPC-speed-skating study, it was not ruled out that an increase in [THbMb] might have been accountable for the enhanced [HHbMb] for the middle section of the race in sprint athletes. In fact, sprinters typically present a high proportion of type II fibers (which are displaying a lower microvascular O_2_ partial pressure; McDonough et al., 2005; Cleland et al., 2012; Paradis-Deschênes et al., 2016) and, thus, an increase in blood volume is more likely to lead to higher O_2_ extraction in these conditions. However, since this concomitant response was not observed in the other sections of the race in the RIPC-speed skating study and considering the results of the present investigation, available data rather suggests that COMBO increased [HHbMb] *per se.*

Importantly, while RIPC was found to accentuate deoxygenation (Barbosa et al., 2015; Paradis-Deschênes et al., 2016) and accelerate deoxygenation dynamics (Kido et al., 2015; Tanaka et al., 2016) in some studies, Cheng et al. (2013) reported that the addition of IMW to a whole-body warm-up significantly attenuates deoxygenation during a high-intensity intermittent sprint test. Therefore, in that perspective, interference in the physiological response of the two used preconditioning strategies cannot be excluded.

### Warm-Up Protocol, Local Hemodynamic, and Performance Enhancement

A priming exercise was found to enhance tolerance to high-intensity exercise with a concomitant significant increase in the [HHbMb] primary amplitude (Bailey et al., 2009), a physiological response that is similar to that observed after RIPC/IPC in some contexts (Kido et al., 2015; Tanaka et al., 2016). However, studies demonstrating IPC/RIPC as ergogenic reported that performance changes were associated with varied deoxygenation patterns [attenuated (Kido et al., 2015; Patterson et al., 2015), accentuated (Barbosa et al., 2015; Paradis-Deschênes et al., 2016), and accelerated dynamics (Kido et al., 2015; Tanaka et al., 2016)]. Moreover, an attenuated muscular deoxygenation was observed after IMW during submaximal and high-intensity intermittent sprint cycling, but this response was not accompanied by an improved performance (Cheng et al., 2013). In long-track speed skating, an enhanced deoxygenation was observed during the gliding and push-off phases of the skating cycle (Hettinga et al., 2016). Although this accentuated deoxygenation may intuitively seem to be deleterious to skating performance, its acute impact on performance remains equivocal from a physiological perspective. Hence, studies investigating the impact of changes in muscle deoxygenation on muscle functioning and performance are still required.

Kjeld et al. (2014) reported performance improvements in three tests after IPC, however, importantly, the least-improved test (1 vs. 8 and 17%) was the only one preceded with a warm-up and priming exercises. In the current investigation, all athletes reported having integrated intense priming exercises to their warm-up routine (**Table 1**). Therefore, since physical activity may elicit a similar preconditioning response to RIPC/IPC (Lalonde et al., 2015; Salvador et al., 2015), it is plausible that the effect of RIPC on performance and local metabolism are limited in field studies that investigate elite athletes who typically perform complete warm-up routines (McGowan et al., 2015; Richard and Billaut, 2018).

### Limitations and Perspectives

These results suggest that COMBO has no practical ergogenic effect on exercise performance in elite speed skaters; therefore the practical applications of combining ischemic preconditioning with IMW appear limited considering the logistical efforts associated with using these techniques in the field. That said, COMBO should be investigated in the context of short-track speed skating since this sport induces a greater ischemic stress compared to long-track speed skating and might therefore benefit from this combination to a greater extent (Hettinga et al., 2016).

Remote ischemic preconditioning treatments were practiced 48, 24, and 1 h before trials, which falls within both the first and the late conditioning phases (Berger et al., 2015). Since ergogenic effects of RIPC/IPC have been observed within a very short time frame (5- to 10-min) (de Groot et al., 2010; Paradis-Deschênes et al., 2016), and up to 8 h (Lisbôa et al., 2017) with respect to the first phase of conditioning, and considering the scarcely studied impact of the second window of condition in sport settings (Seeger et al., 2017), we cannot rule out the influence of the timing on the current speed-skating performances.

To promote a realistic context, the athletes were not blinded about their performance data (timing system). However, the unchanged rates of perceived exertion suggest that the effort was similar between conditions. The limited pacing possibilities in such a sprint distance also minimize the potential impact of this limitation. The current investigation was conducted during a training camp. While very similar training conditions preceded both testing days, this context may implicate varied fatigue levels among athletes (17 training sessions in 14 days with six intense training sessions including the trials). Nevertheless, some of those limitations contribute to mimicking a more genuine and realistic competitive environment and seldom differences in sensations between the two races were reported by the athletes in post-facto interviews (one minor technical issue). Similarly, although studying the effect of a combination of preconditioning strategies contributes to ensuring a very field-specific study context and increases the likelihood of applicability of the results, this experimental model does not permit to assess the individual impact of each technique (chronic RIPC vs. acute RIPC vs. IMW).

The small sample size is a limitation in this study. However, investigation of elite athletes is underrepresented in the literature, and the strength of the sample and originality of the findings reside in its specificity.

## Conclusion

The results of this investigation suggest that the combination of chronic RIPC with IMW may have a limited effect as a strategy to improve exercise performance and gain a competitive advantage in elite long-track speed skaters. While COMBO led to small and moderate increases in leg muscle deoxygenation, this did not affect performance. Thus, the relationship between changes in muscle oxygenation and performance remains equivocal, at least in speed skating, and further studies will need to better understand the impact of such peripheral changes. In the perspectives of training beyond PyeongChang 2018, it is certain that athletes will continue combining ergogenic aids and techniques in the hope of enhancing their physiological responses, and it is critical that research in the sport sciences keeps up with such practices and assess the efficacy of these interactions.

## Data Availability Statement

All relevant data is contained within the manuscript but ungroup individual performance data are not publicly available for ethical reasons; publishing them may render possible to recognize the athletes.

## Author Contributions

PR and FB conceived and designed the research, interpreted the results, and edited and revised the manuscript for approval of the final version. PR collected and analyzed the data, and drafted the manuscript.

## Conflict of Interest Statement

The authors declare that the research was conducted in the absence of any commercial or financial relationships that could be construed as a potential conflict of interest.
